# Cost-Effectiveness Analysis of Triple Combination Preparations in the Treatment of Moderate-to-Severe Chronic Obstructive Pulmonary Disease

**DOI:** 10.3389/fpubh.2021.713258

**Published:** 2021-07-28

**Authors:** Yikang Zhou, Enwu Long, Qian Xu, Lei Wang, Xuehua Jiang, Ming Hu

**Affiliations:** ^1^West China School of Pharmacy, Sichuan University, Chengdu, China; ^2^Sichuan Academy of Medical Sciences, Sichuan Provincial People's Hospital, Chengdu, China; ^3^China National Health Development Research Center, National Health Commission of the People's Republic of China, Beijing, China; ^4^School of Pharmaceutical Science and Technology, Tianjin University, Tianjin, China

**Keywords:** chronic obstructive pulmonary disease, economic evaluation, cost-effectiveness analysis, triple combination preparations, Markov model

## Abstract

**Objectives:** This study analyzed the long-term cost-effectiveness of fluticasone/umeclidinium/vilanterol triple combination (FF/UMEC/VI) vs. budesonide/formoterol double combination (BUD/FOR) in the treatment of moderate-to-severe chronic obstructive pulmonary disease (COPD) and provides evidence for COPD treatment decisions.

**Methods:** From the perspective of the healthcare system, a Markov model was established that consists of four states—stable period, non-severely deteriorating period, severely deteriorating period, and death—according to real-world COPD progression. The model period comprises 6 months, with a cycle length of 14 years. The initial state, transition probabilities, costs, and utility data were collected from the FULFIL trial, published literature, hospital record surveys, and *China Health Statistics Yearbook*. The discount rate was 5%, and the threshold was set as the Chinese per capita GDP in 2020 (¥72,447). The cost, utility, transition probabilities, and discount rate were calculated through TreeagePro11 software. The results were analyzed *via* one-way factor analysis and probability sensitivity analysis.

**Results:** The baseline study shows that the 14-year treatment for FF/UMEC/VI and BUD/FOR groups are ¥199,765.55 and ¥173,030.05 with effectiveness at 8.54 quality-adjusted life years (QALYs) and 7.73 QALYs, respectively. The incremental cost-effectiveness ratio is ¥33,006.80/QALY, which is below the threshold. A tornado diagram of a one-way sensitivity analysis shows that the top three factors that affected the results are the non-severe deterioration rates of FF/UMEC/VI, the cost of FF/UMEC/VI and the non-severe deterioration rates of BUD/FOR. Probabilistic sensitivity analysis shows that FF/UMEC/VI (compared to BUD/FOR) can be made cost-effective under the willingness-to-pay (WTP) threshold (¥38,000). Furthermore, the likelihood of cost-effectiveness increases with a higher WTP.

**Conclusions:** Compared with the double combination (BUD/FOR), the triple combination (FF/UMEC/VI) is more cost-effective under the Chinese per capita GDP threshold.

## Introduction

Chronic obstructive pulmonary disease (COPD) is a kind of chronic bronchitis and/or emphysema characterized by airflow obstruction that can further develop into the common chronic diseases of pulmonary heart disease and respiratory failure. COPD is related to an abnormal inflammatory reaction to harmful gases and particles and has high disability and mortality rates (1). It is also one of the most rapidly growing causes of death in developed countries. While the mortality rates of cardiovascular and cerebrovascular diseases have fallen sharply during the period from 1990 to 2010, the mortality rate associated with COPD has increased by 163% (2). In China, more than one million COPD patients die every year (3), and COPD is the fourth leading cause of death worldwide (4).

COPD severely affects quality of life and imposes substantial burden on patients, their families, and society, which in turn severely impacts public health and the economy (5). The results of a survey on disability adjusted life years (DALYs) due to a total of 306 diseases in 188 countries showed that DALY loss due to COPD ranked 7th in the world in 2013, 6th in economically developed countries, and 10th in developing countries (6). Data from the World Bank and the World Health Organization indicates that by 2020, the economic burden of COPD will rank it as the 5th most expensive disease in the world. *The New European Lung White Book* points out that the direct and indirect economic burden caused by COPD is expected to exceed €100 billion in 2013, which is significantly higher than €3.86 billion in 2003 (7). In 2006, The Chinese Center for Disease Control and Prevention noted that COPD ranked second in terms of burden from chronic diseases in China (8). There are currently 100 million patients with COPD in China, and COPD has become the third most common chronic disease in China after hypertension and diabetes mellitus. The prevalence rates of COPD among adults over 20, 40, and 60 years old are 8.6, 13.7, and over 27%, respectively (9). According to the 2015 Global Burden of Disease Report, COPD ranked third in terms of causes of disease death in China after cerebrovascular disease and ischemic heart disease, and the number of deaths accounted for 9.7% of overall deaths (10).

The 2020 Global Initiative for Chronic Obstructive Lung Disease (GOLD) states that COPD treatment goals are to prevent disease progression, relieve symptoms, improve exercise capacity, improve health status, prevent and treat complications, prevent exacerbations, and reduce mortality (11). The current main treatments for COPD are drug and non-drug interventions. Drug therapy is an essential method for addressing COPD, as this can help to prevent and control symptoms, reduce the frequency of acute exacerbations, and improve the quality of life and exercise tolerance of patients. The “China Chronic Obstructive Pulmonary Diagnosis and Treatment Guidelines (2013),” “NICE: Chronic Obstructive Pulmonary Disease in over 16s: Diagnosis and Management,” and other guidelines mention that drug treatment can relieve symptoms experienced by COPD patients and improve their lung function. Furthermore, drug treatment plays an essential role in improving patients' quality of life (12, 13).

Patients with moderate-to-severe COPD often need to use a combination of drugs with three different mechanisms of inhaled corticosteroid/long-acting muscarinic antagonist/long-acting b2-agonist(ICS/LAMA/LABA) at the same time. GOLD also recommends inhaled triple-drug therapy (ICS/LAMA/LABA) for COPD patients with persistent symptoms and exacerbations (11). However, although these treatment options with various underlying mechanisms are widely used, few randomized controlled trials have shown sustained advantages in lung function and patient-reported outcome indicators compared with using ICS/LABA alone. In addition, the triple treatment plan requires the use of more than two inhalation devices, which can lead to dosing errors. Therefore, there is an urgent need to improve the approach used to administer the triple combination of ICS/LAMA/LABA.

At present, there are alternative triple combinations for treating COPD, including fluticasone/umeclidinium/vilanterol (FF/UMEC/VI) and budesonide/glycopyrrolate/formoterol. However, there is insufficient economic evidence to support the use of these triple combinations. Here we developed a Markov model to simulate and analyzed the long-term cost-effectiveness of FF/UMEC/VI and the double combination budesonide/formoterol (BUD/FOR) based on the published results of the Phase III FULFIL study of a COPD triple combination (FF/UMEC/VI) (14). The results provide evidence to support a reasonable treatment strategy for COPD.

## Materials and Methods

### Analytical Method

A Markov model was constructed to evaluate the cost and output of an intervention group and a control group.

### Intervention

This study was based on a Phase III, randomized, double-blind, double-simulation, parallel-group, multi-center FULFIL study (GSK: CTT116853; ClinicalTrials.gov: NCT02345161). In the FULFIL study, patients with COPD were defined as being in Gold Initiative for Chronic Obstructive Lung Disease group D (Appendix II) (11). It was conducted over a total of 24 weeks and involved 1,810 patients with a mean age of 63.9 years.

In the intervention group (*n* = 911), FF/UMEC/VI, patients mainly used a single ELLIPTA inhaler to deliver once-daily FF/UMEC/VI 100 μg/62.5 μg/25 μg inhalation powder; in the stable period, non-severely deteriorating period, and severely deteriorating period, drugs and oxygen therapy were added to address the patients' symptoms.

In the control group (*n* = 899), BUD/FOR, patients mainly used Turbuhaler to deliver twice-daily BUD/FOR 400 μg/12 μg; in the stable period, non-severely deteriorating period, and severely deteriorating period, drugs and oxygen therapy were added to address the patients' symptoms.

### Markov Model Structure

**Figure 1:**
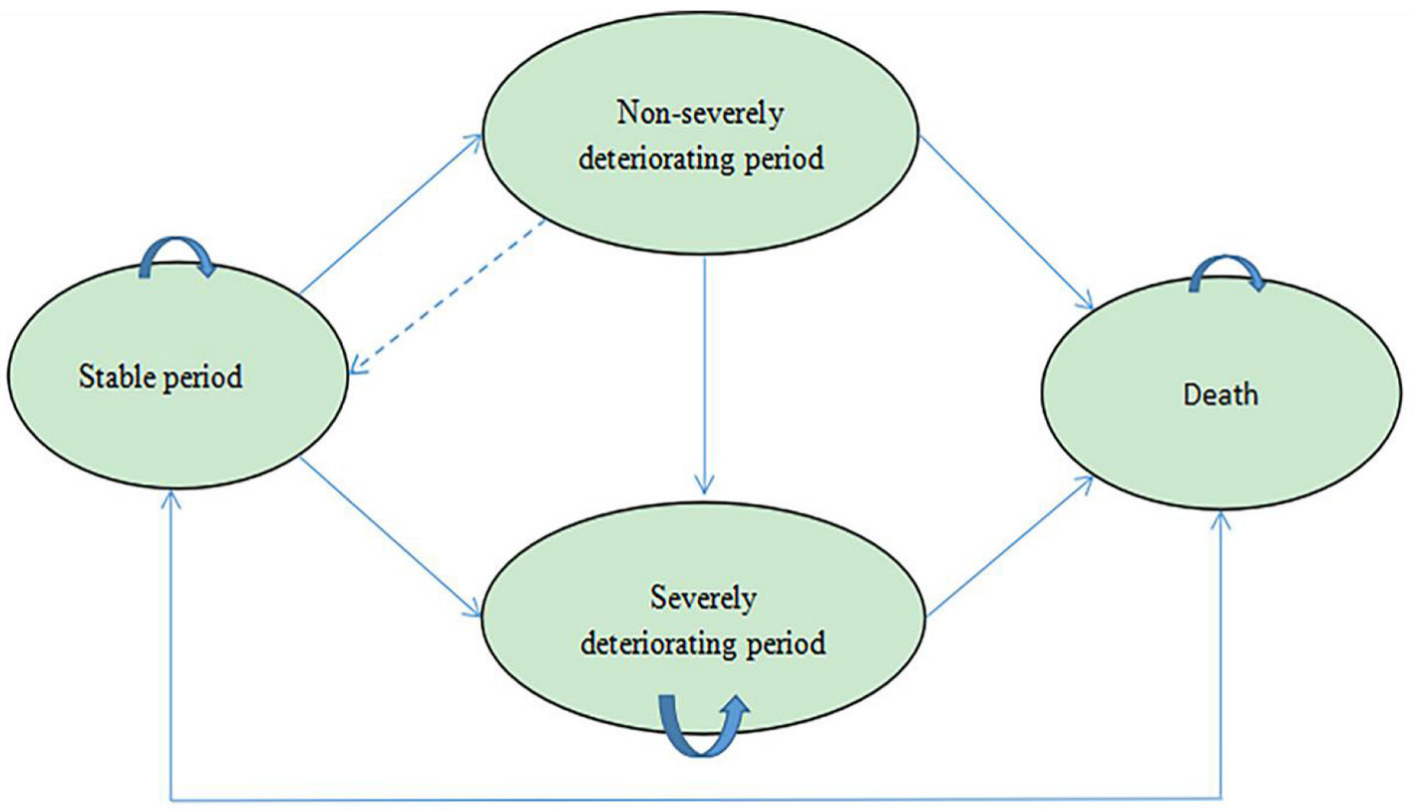
Model structure.

Decision analysis software (TreeAge Pro 2011, Williamstown, MA) was used to establish a Markov model to realize the calculation process, which consisted of four states (Figure 1)—stable period, non-severely deteriorating period, severely deteriorating period, and death—according to the real-world disease progression of COPD. The degree of deterioration was divided according to whether additional medical resources were needed: patients in the non-severely deteriorating period only need to have their medication adjusted according to their general practitioner's instructions, while patients in severe exacerbation stages need to be hospitalized for treatment. According to the FULFIL test, the cycle length was 6 months.

### Cost Parameters

From the perspective of the healthcare system, all direct medical costs were included in this study, which mainly include drug costs, diagnosis costs and maintenance treatment costs in the stable and deteriorating periods. The costs of medicines were taken from the average bid price of 2020 in China by querying the bid information of www.yaozh.com.^1^ The costs of diagnosis and laboratory tests were derived from a real-world clinical data survey. The maintenance treatment cost was based on a previous COPD pharmacoeconomic study (15). The maximum and minimum values of the parameters were based on the basic value ±10% of all costs and the mean value of that cost ±10% (Table 1).

**Table 1:** Cost parameters.

**Project Fees (¥)**		**Base value**	**Minimum**	**Max**	**Distribution**	**References**
Medicine	FF/UMEC/VI^a^	5,400.00	4,860.00	5,940.00	Gamma: α 606 β 0.1	(see text footnote 1)
	BUD/FOR^b^	2,695.38	2,425.84	2,964.92	Gamma: α 84 β 0.04	(see text footnote 1)
General practitioner		26.00	23.40	28.60	Gamma: α 4 β 0.08	Clinical data
Laboratory test	Spirometry	80.00	72.00	88.00	Gamma: α 384 β 6	Clinical data
Maintenance treatment	Stable period	319.26	287.34	351.19	Gamma: α 384 β 1	(15)
	Non-severe deterioration period	567.01	510.31	623.71	Gamma: α 384 β 0.7	(15)
	Severe deterioration period	20,610.84	18,549.76	22,671.92	Gamma: α 384 β 0.01	(15)

a *Fluticasone/umeclidinium/vilanterol triple combination*.

b *Budesonide/formoterol double combination*.

### Effectiveness Parameters

Quality-adjusted life years (QALYs) were used as the effect indicators of this model. The utility value of each state was extracted and calculated based on previous literature. Non-severe deterioration will cause a loss of 0.01/year of health utility, severe deterioration will cause a loss of 0.04/year of health utility, and the health utility of death is 0 (Table 2). The utility of the stable phase was converted from the St George's respiratory questionnaire (SGRQ) score of the FULFIL study population using the published formula (16) (Equation 1).

(1) $$\begin{array}{l} U\;=\;0.9617-(0.0013\;\times \;SGRQ\;total)-(0.0001\;\times \;SGRQ\;total^{2})\\ \;+(0.0231\;\times \;male) \end{array}$$

**Table 2:** Model utility parameters.

**Status**	**Base value**	**Minimum**	**Max**	**Distribution**	**References**
Stable period	0.65	0.64	0.66	Beta: α 5,680 β 3,058	(14)
Non-severe deterioration loss/year	0.01	0.00	0.024	Beta: α 4 β 632	(16)
Serious deterioration loss/year	0.04	0.024	0.06	Beta: α 20 β 954	(16)
Death	0				

### Transition Probability

The transition probability mainly came from clinical trials, the literature, and official Chinese databases. The annual natural mortality rate of the population was obtained from the “National Death Population Status by Age and Sex (2017–2018)” and was transformed into the natural mortality rate of patients during the study period (Appendix I). The transition probability of patients from a stable period to non-severe and severe deterioration was calculated from the deterioration of the patient population in the FULFIL study. The mortality rate of severely deteriorated patients comes from the “2008 National Chronic Obstructive Pulmonary Disease Audit in the UK” (17) (Table 3).

**Table 3:** Transition probability.

**Status**			**Base value**	**Minimum**	**Max**	**Distribution**	**References**
Stable-	Non-severe	FF/UMEC/VI	0.1195	0.1076	0.13145	Beta: α 338 β 2,492	(14)
		BUD/FOR	0.1563	0.1407	0.17193	Beta: α 324 β 1,749	(14)
	Severe	FF/UMEC/VI	0.0148	0.0133	0.01628	Beta: α 4 β 251	(14)
		BUD/FOR	0.0264	0.0238	0.02904	Beta: α 374 β 13,793	(14)
	Death		P_D: Natural mortality				
Non-severe-	Stable		0.5345	0.4811	0.58795	Beta: α 178 β 155	(14)
	Severe		0.4655-P_D				
	Death		P_D: Natural mortality				
Severe-	Severe		1-P_D				
	Death		0.0770	0.0693	0.0847	Beta: α 354 β 4,250	(17)

### Willingness-to-Pay Threshold

Concerning the reference threshold value of Chinese medical insurance drug negotiations in recent years, this study set one-time per capita GDP as the threshold value for willingness-to-pay (WTP). According to official data from the National Bureau of Statistics, the 2020 per capita GDP was ¥72,447 in China.^2^ When the incremental cost-effectiveness ratio (ICER) is lower than ¥72,447/QALY, the treatment scheme is considered cost-effective.

### Cycle Length and Discount Rate

The average life expectancy in China in 2019 (77.3 years), minus the average age of subjects included in the FULFIL study (63.9 years), was used as the basis for the model's operating time limit. Therefore, the study set 28 cycles to simulate the long-term cost-utility of patients 14 years after treatment. The baseline discount rate was 5% according to the *Chinese Pharmacoeconomic Evaluation Guidelines and Guide (2020) Edition* (18).

### Sensitivity Analysis

#### One-Way Sensitivity Analysis

In the one-way sensitivity analysis, the value was assigned with a varied range of ±10% of the basic value; the discount rate's variation range was 0–8%. The key factors that had the most significant impacts on the research results were obtained through one-way sensitivity analysis. The results are presented in a one-way analysis table and an ICER tornado chart.

#### Probabilistic Sensitivity Analysis

Monte Carlo simulation was used for probabilistic sensitivity analysis (PSA). The research results for different parameters changing simultaneously were obtained through 1,000 simulations. Based on this stochastic simulation method, the research results from simultaneous changes in multiple parameters and the influence of threshold changes on decision-making were analyzed. The results are presented in Monte Carlo simulation scatter plots, cost-effectiveness acceptability curves, and other charts.

## Results

### Baseline Results

FULFIL demonstrated clinically meaningful and statistically significant improvements at Week 24 in SGRQ total scores. There was a difference of −2.2 (95% CI −3.5, −1.0; *p* < 0.001) in change from baseline in SGRQ total score for FF/UMEC/VI vs. BUD/FOR (Appendix II) (14).

Following cohort simulation with the Markov model, the cumulative costs of the FF/UMEC/VI treatment for each COPD patient was determined to be ¥199,765.55, and total QALY gains were 8.54. The costs of BUD/FOR were ¥173,030.05, and the QALY gains were 7.73. The FF/UMEC/VI group paid ¥26,735.50 more in costs compared to the BUD/FOR group but gained 0.81 extra QALYs. The ICER was ¥33,006.80/QALY, which was less than the set threshold of ¥72,447/QALY, indicating that the FF/UMEC/VI treatment is more cost-effective than BUD/FOR (Table 4).

**Table 4:** Basic analysis results.

**Project**	**Cumulative cost(¥)**	**Cumulative effectiveness (QALYs)**	**Incremental cost**	**Incremental effectiveness**	**ICER (¥/QALY)**
BUD/FOR^a^	173,030.05	7.73			
FF/UMEC/VI^b^	199,765.55	8.54	26,735.50	0.81	33,006.80

a *Fluticasone/umeclidinium/vilanterol triple combination*.

b *Budesonide/formoterol double combination*.

### One-Way Sensitivity Analysis

**Figure 2:**
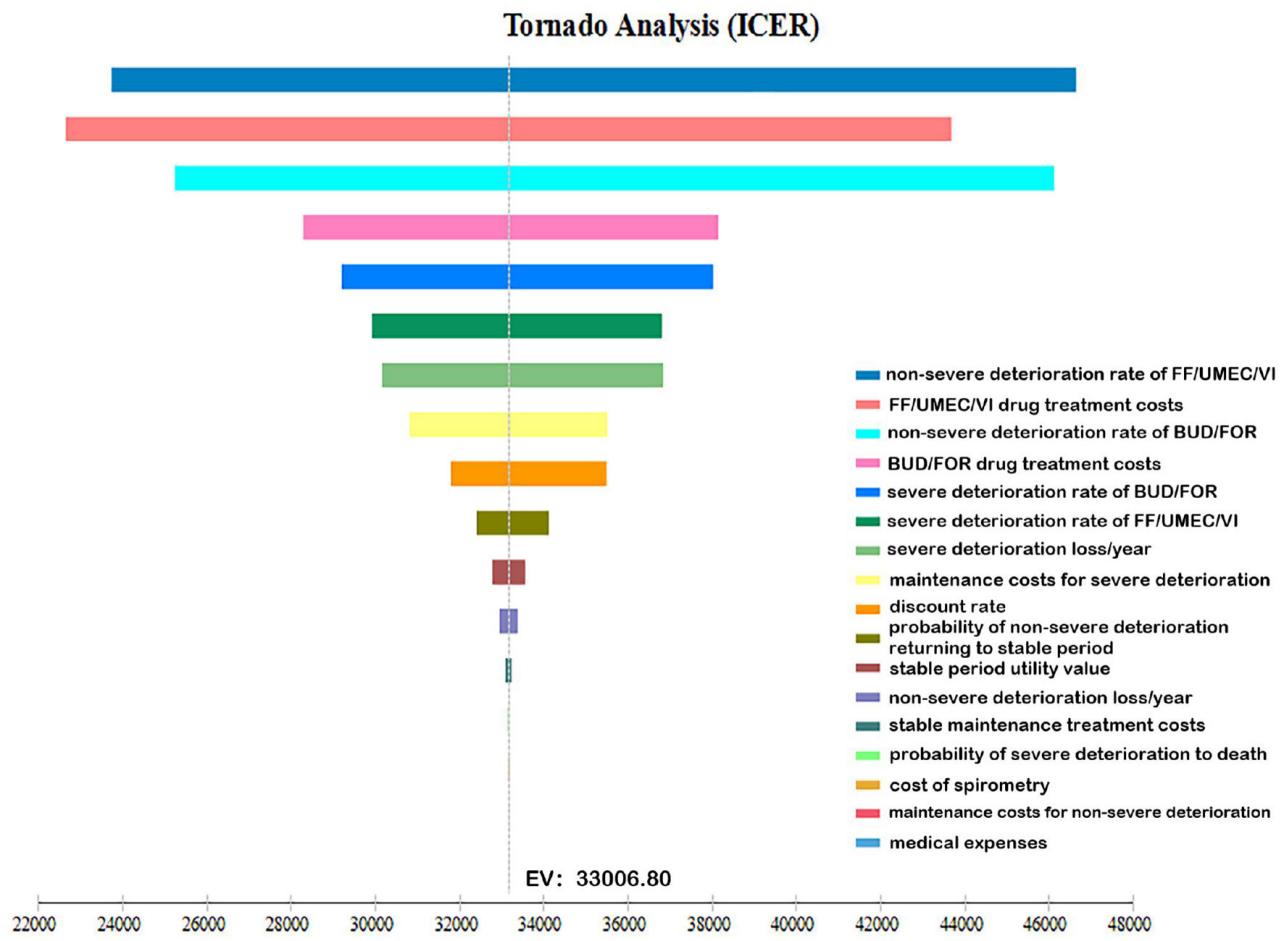
One-way sensitivity analysis tornado diagram. The influencing factors from highest to lowest are: A: non-severe deterioration rate of FF/UMEC/VI; B: FF/UMEC/VI drug treatment costs; C: non-severe deterioration rate of BUD/FOR; D: BUD/FOR drug treatment costs; E: severe deterioration rate of BUD/FOR; F: severe deterioration rate of FF/UMEC/VI; G: severe deterioration loss/year; H: maintenance treatment costs for severe deterioration period; J: discount rate; K: probability of non-severe deterioration returning to stable period; L: stable period utility value; M: non-severe deterioration loss/year; N: stable maintenance treatment costs; O: probability of severe deterioration returning to death; P: cost of spirometry; Q: maintenance treatment costs for non-severe deterioration; R: medical expenses.

A one-way sensitivity analysis was performed with the changes in cost, utility, transition probability, and discount rates of the two drug treatment options. The ICER tornado chart is shown in Figure 2. The top three factors that affected the results are the non-severe deterioration rates of FF/UMEC/VI, the cost of FF/UMEC/VI and the non-severe deterioration rates of BUD/FOR.

### PSA

**Figure 3:**
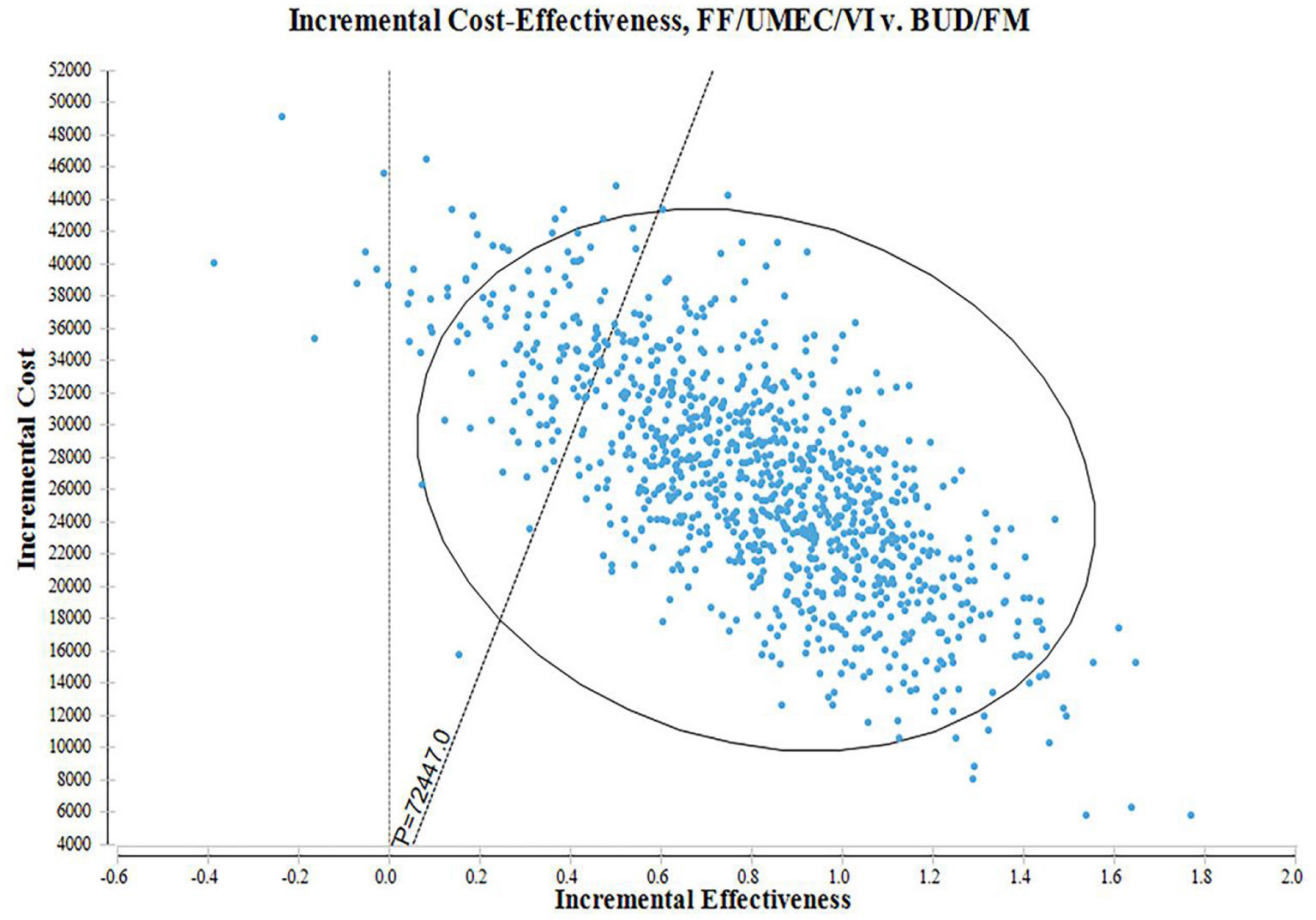
Monte Carlo simulation pseudo-scatter plot. Dotted line: WTP = ¥72,447/QALY.

**Figure 4:**
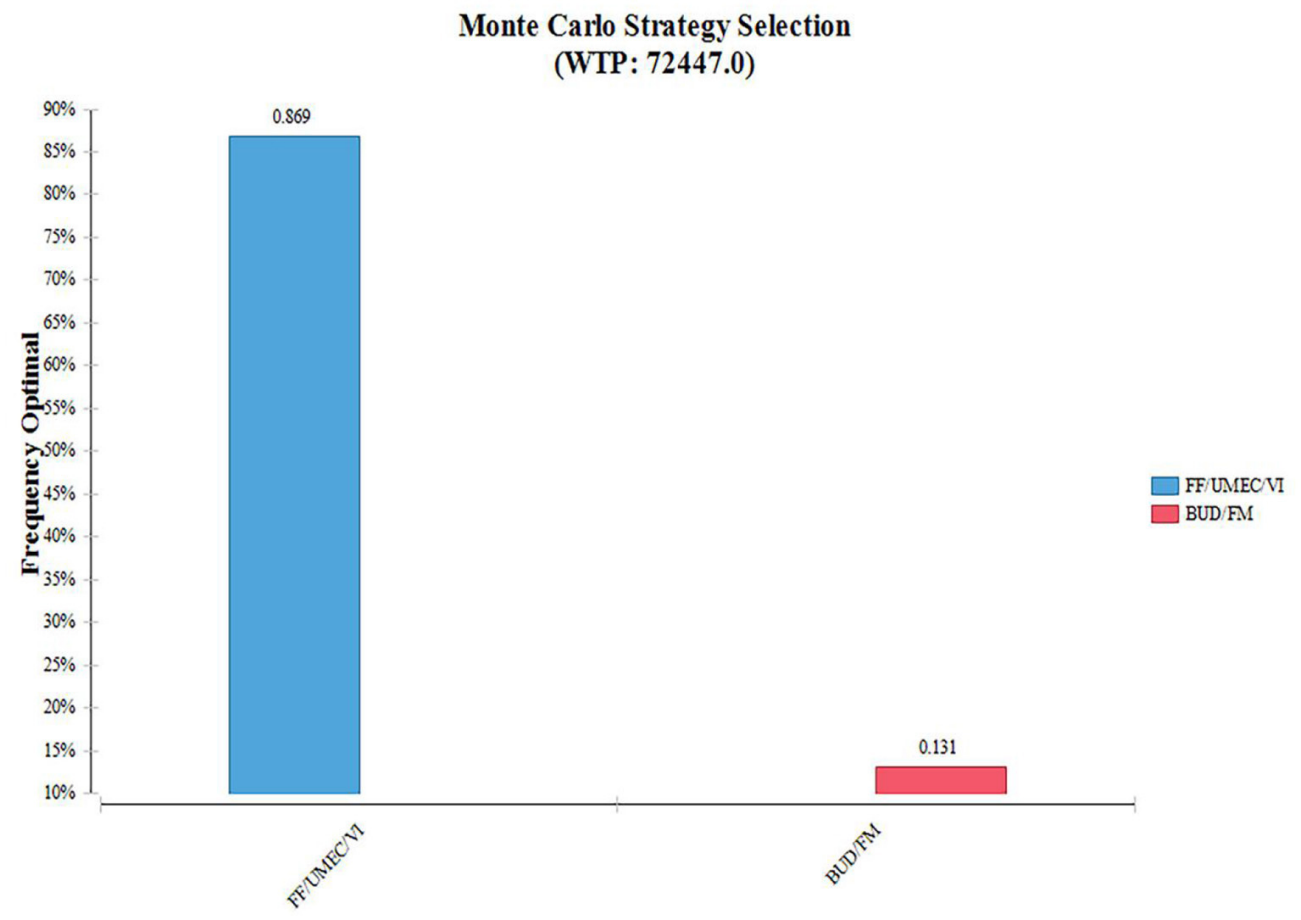
Cumulative cost-acceptable probability of effect. FF/UMEC/VI, fluticasone/umeclidinium/vilanterol; BUD/FOR, budesonide/formoterol.

According to the distribution of various parameters, Monte Carlo simulation was used to simulate the research results 1,000 times. The ICER scatter plot is shown in Figure 3. A total of 86.9% points fell below the threshold line in Figure 4, indicating that in 1,000 simulations, FF/UMEC/VI had a higher probability of offering economic advantages over BUD/FOR.

**Figure 5:**
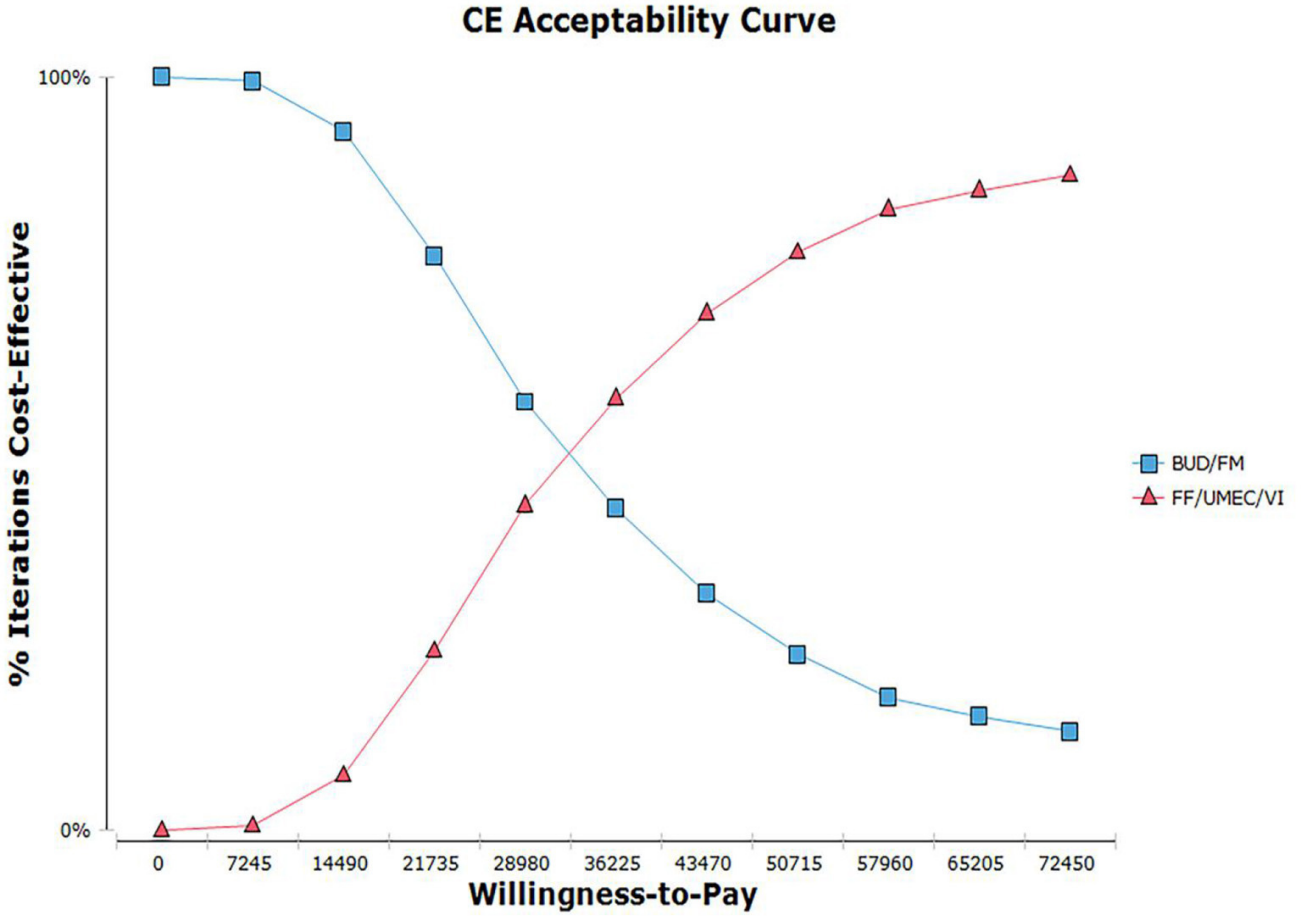
Cost-effectiveness acceptable curve plotting. Willingness-to-pay vs. iterations cost-effective.

To further illustrate the relationship between the cost-effectiveness threshold change a cost-effectiveness acceptability curve (CEAC) was drawn, as shown in Figure 5. According to the CEAC, when WTP was higher than about ¥38,000, FF/UMEC/VI was more likely to be economically advantageous than BUD/FOR.

## Discussion

A variety of COPD treatment drug regimens are currently available, including the most prominent class of bronchodilators and expectorants, corticosteroids, phosphodiesterase-4 inhibitors, and antimicrobials that target airway obstruction. Among bronchodilators, single-agent use and the combination of LABA, LAMA, and inhaled corticosteroids are recommended in the COPD related guidelines. The common diphasic combinations are LABA/LAMA (Indacaterol/Glonoprost, Umeclidinium/Vilanterol, etc.), and LABA/ICS (Salmeterol/Tikasone, Budesonide/Formoterol, etc.); inhalation formulations are more widely used in clinical practice. The triple combination system ICS/LAMA/LABA (Budesonide/Glycopyrronium/Formoterol, Fluticasone/ Umeclidinium/Vilanterol, Beclometasone/ Formoterol/Glycopyrronium) is also continuously being developed and produced and is recommended by GOLD and other guidelines for the treatment of moderate-to-severe COPD.

Regarding triple combination preparations, FF/UMEC/VI can significantly reduce the hospitalization rate and all-cause mortality of COPD patients compared with LAMA/LABA preparations (19, 20). Compared with the commonly used ICS/LABA preparation, FF/UMEC/VI can also significantly alleviate COPD symptoms, improve lung function, and reduce acute exacerbations (21). Schroeder showed that although the cost of FF/UMEC/VI is greater than that of BUD/FOR, the benefit of FF/UMEC/VI is higher than it is for BUD/FOR or other double combinations in terms of long-term cost effects (22). Papi and Martinez also showed that other triple combination preparations are advantageous compared to double combinations; for instance, the triple combination Beclometasone/Formoterol/Glycopyrronium is associated with a significantly larger reduction in the rate of moderate-to-severe COPD exacerbations compared to the dual bronchodilator combination of Indacaterol/Glycopyrronium over 52 weeks of treatment. Likewise, Budesonide/Glycopyrronium/Formoterol offers greater benefits in reducing moderate/severe and severe exacerbation rates relative to Glycopyrrolate/Formoterol, even among COPD patients with no history of exacerbations in the prior year (23, 24). Thus, the findings of the present study are in line with those of extant literature.

In addition, the effects of cigarette smoking also need to be taken into account when treating COPD with the appropriate pharmacotherapy. Across the world, cigarette smoking is the most commonly encountered risk factor for COPD (11). Cigarette smokers have a higher prevalence of respiratory symptoms and lung function abnormalities and a greater COPD mortality ratee than non-smokers (25). Smoking cessation can significantly reduce morbidity and mortality associated with COPD in conjunction with pharmacotherapy. China has also taken many strategies in tobacco control: In 2006, China signed the World Health Organization Framework Convention on Tobacco Control (WHO FCTC) to combat the tobacco epidemic; Up to data as of January 2018, 18 cities, including Beijing, Shanghai, Hangzhou, and Guangzhou, have introduced local tobacco control regulations, covering nearly 10% of the country's population.^3^ The “Health China 2030” plan also set specific targets for tobacco control, with a reduction in the smoking rate to 20% of the population by 2030.^4^

Finally, pharmacist-led intervention and Effective use of the inhaler are crucial for COPD patients, too. Abdulsalim showed that Structured pharmacist-led intervention programme can improve medication adherence in COPD patients and effective use of the inhaler. Better medication adherence is associated with decrease in the number of emergency department visits and length of hospital stay among patients with COPD with improved adherence saving medicine costs and clinical pharmacy time (26–28).

Overall, we found that FF/UMEC/VI is more economical than BUD/FOR from the perspective of Chinese healthcare system. The sensitivity analysis results showed that the baseline analysis results are robust. The conclusions of this study are consistent with the GALAXY-COPD joint risk equation model (29). A systematic literature review about COPD showed that annual total societal costs of COPD ranged from $4398 to $23,049 in Japan and $453 to $12,167 in South Korea. There were no domestic comparison estimates for the remaining countries (Singapore: $2700; Taiwan: $4000; China: $3942; and Thailand: $1105) (30). This was consistent with the findings of our study that COPD imposes a significant financial burden on society, the health insurance segment, patients, etc.

In this study, a classic Markov model was constructed based on the disease outcome of COPD patients in the real world. Due to issues with data availability and to ensure experimental rigor, this study did not use the 1% forced expiratory volume value recommended by the commonly used GOLD guidelines and other COPD studies to classify the disease into several states (e.g., mild, moderate, severe, and extremely severe). Instead, the stable and deteriorating periods simulate the patient's disease progression. Additional real-world data and clinical findings are needed to support methodological refinement for the economic evaluation of COPD treatment options.

This study is subject to several limitations. First, the Markov model was based on a simulation of the patient's ideal state based on certain assumptions. For instance, it was assumed that FF/UMEC/VI and BUD/FOR had the same remission rate for non-severe deterioration, and the mortality rate of non-severe deterioration was based on natural mortality, which impacts extrapolation of the results to a certain extent. Second, the utility value was derived from calculating the formula, and this study only simulated the loss of utility value in patients with worsening conditions, without taking into account the increase in utility value brought about by improvement of the condition. Therefore, we may have underestimated the quality of health output. Third, there are currently no utility value measurement studies for the Chinese population of COPD patients with acute exacerbation and stable disease. It is thus recommended that further research on the quality of life of COPD patients be carried out in future. Fourth, more attention needs to be paid to the impact of long-term glucocorticoid use on increasing the risk of pneumonia and the prognosis of patients with proper inhaler use, these can affect the outcome and cost of treatment for patients.

## Conclusion

Compared with the double-combination treatment option for COPD, BUD/FOR, our results showed that the triple combination FF/UMEC/VI was cost-effective under the threshold of Chinese per capita GDP. Simulations over 14 years of the model showed that FF/UMEC/VI can increase patient benefits and save on health resources. The 2020 edition of the *Global Chronic Obstructive Pulmonary Disease Initiative* recommends that patients with moderate-to-severe stable stages choose the ICS/LAMA/LABA triple combination for treatment. However, additional real-world data from the Chinese population and more accurate cost data are needed to test this hypothesis.

## Data Availability Statement

The original contributions presented in the study are included in the article/Supplementary Material, further inquiries can be directed to the corresponding author/s.

## Ethics Statement

Written informed consent was not obtained from the individual(s) for the publication of any potentially identifiable images or data included in this article.

## Author Contributions

MH conceived and designed the study. YZ, EL, QX, LW, and XJ contributed materials and collected the data. YZ and EL analyzed the data. YZ, EL, QX, and LW wrote the manuscript. YZ and EL contributed equally to this manuscript. All authors contributed to the article and approved the submitted version.

## Conflict of Interest

The authors declare that the research was conducted in the absence of any commercial or financial relationships that could be construed as a potential conflict of interest.

## Publisher's Note

All claims expressed in this article are solely those of the authors and do not necessarily represent those of their affiliated organizations, or those of the publisher, the editors and the reviewers. Any product that may be evaluated in this article, or claim that may be made by its manufacturer, is not guaranteed or endorsed by the publisher.

## Footnotes

^1^YAOZH [EB/OL]. Available online at: https://www.yaozh.com/ (accessed December 30, 2020).

^2^National Bureau of Statistics (2020). Available online at: https://data.stats.gov.cn/search.htm?s=%E4%BA%BA%E5%9D%87GDP (accessed December 30, 2020).

^3^Report on the Project to Accelerate Tobacco Control Legislation in China. Chinese Preventive Medical Association.

^4^The State Council of PRC [Online]. Available online at: http://www.gov.cn/xinwen/2016–10/25/content_5124174.htm (accessed October 25, 2016).

## References

[1] Abdallah F Kacem M Bachouch I Belloumi N Fenniche S. Pharmacological treatment of stable Chronic Obstructive Pulmonary Disease: where do we stand? In: ERS International Congress 2018 Abstracts. Paris: Paris Expo Porte de Versailles (2018).

[2] Yang G Wang Y Zeng Y Gao GF Liang X Zhou M. Rapid health transition in China, 1990–2010: findings from the Global Burden of Disease Study 2010. Lancet. (2013) 381:1987–2015. 10.1016/S0140-6736(13)61097-1 23746901 PMC7159289

[3] Jiang HE Yang X-K Song F-y Chen X-c Min K Li Z-f Ban W-f. Mortality trend of chronic obstructive pulmonary disease, Qiannan, 1998–2017. Modern Prevent Med. (2019) 46:2470–3. Available online at: https://kns.cnki.net/kcms/detail/detail.aspx?dbcode=CJFD&dbname=CJFDLAST2019&filename=XDYF201913039&v=qRZsO58PZZRbXwEgyZV7PWD3qvNcHX7Y%25mmd2BFjkyqpbxIqbQjLwJp8sw%25mmd2FIb3L7e%25mmd2Bz8G

[4] Vos T Barber RM Bell B Bertozzi-Villa A Biryukov S Bolliger I. Global, regional, and national incidence, prevalence, and years lived with disability for 301 acute and chronic diseases and injuries in 188 countries, 1990–2013: a systematic analysis for the Global Burden of Disease Study 2013. Lancet. (2015) 2015:4. 10.1016/S0140-6736(15)60692-4 26063472 PMC4561509

[5] Hongxia Wu DC Wei J. The disease burden of chronic obstructive pulmonary disease and its influencing factors. Clin Meta. (2015) 30:1063–6. 10.3969/j.issn.1004-583X.2015.09.030 Available online at: https://xueshu.baidu.com/usercenter/paper/show?paperid=a3d38ed3e6758260b4702b66e016970d&site=xueshu_se32368022

[6] Murray C Barber RM Foreman KJ Ozgoren AA Abdallah F Abera SF. Global, regional, and national disability-adjusted life years (DALYs) for 306 diseases and injuries and healthy life expectancy (HALE) for 188 countries, 1990–2013: quantifying the epidemiological transition. Lancet. (2015) 386:2145–91. 10.1016/S0140-6736(15)61340-X 26321261 PMC4673910

[7] Gibson GJ Loddenkemper R Lundback B Sibille Y. Respiratory health and disease in Europe: the new European Lung White Book. Eur Respiratory J. (2013) 42:559–63. 10.1183/09031936.00105513 24000245

[8] Bureau of Disease Control and Prevention Ministry of Health of the People's Republic of China National Health Commission of the People's Republic of China. Report on Chronic Disease in China. Available online at: http://www.gov.cn/gzdt/2006–05/12/content_279061.htm (accessed May 12, 2006).

[9] Wang C Xu J Yang L Xu Y Zhang X Bai C. Prevalence and risk factors of chronic obstructive pulmonary disease in China (the China Pulmonary Health [CPH] study): a national cross-sectional study. Lancet. 391:1706–17. 10.1016/S0140-6736(18)30841-9 29650248

[10] Mortality G Collaborators CoD. Global, regional, and national age-sex specific all-cause and cause-specific mortality for 240 causes of death, 1990–2013: a systematic analysis for the Global Burden of Disease Study 2013. Lancet. (2015) 385:117–71. 10.1016/S0140-6736(14)61682-2 25530442 PMC4340604

[11] Global Initiative for Chronic Obstructive Lung Disease. Global Strategy for the Diagnosis, Management, and Prevention of Chronic Obstructive Pulmonary Disease 2020 Report. Available online at: https://goldcopd.org/gold-reports/ (accessed November 13, 2019).

[12] Chronic Obstructive Pulmonary Disease (COPD) Group of the Chinese Medical Association Respiratory Diseases Branch. Guidelines for Diagnosis and Treatment of Chronic Obstructive Pulmonary Disease (2013 Revised Edition). Guangzhou (2013).

[13] National Institute for Health and Care Excellence. Chronic Obstructive Pulmonary Disease in Over 16s: Diagnosis and Management. (2018). Available online at: https://www.nice.org.uk/guidance/ng115/ (accessed December 05, 2018). 31211541

[14] Zheng J Zhong N Wang C Huang Y Chen P Wang L. The efficacy and safety of once-daily fluticasone furoate/umeclidinium/vilanterol versus twice-daily budesonide/formoterol in a subgroup of patients from China with Symptomatic COPD at Risk of Exacerbations (FULFIL Trial). Copd J Chronic Obstruct Pulmonary Dis. (2018) 2018:1–7. 10.1080/15412555.2018.1481022 30265816

[15] Fan C. The cost-effectiveness analysis of indacaterol versus tiotropium in chinese medical cost setting. Drug Evaluation. (2016) 13:34–9. 10.3969/j.issn.1672-2809.2016.01.008

[16] Mölken DMPMHR-v Hoogendoorn M Lamers LM. Holistic preferences for 1-year health profiles describing fluctuations in health: the case of chronic obstructive pulmonary disease. Pharmacoeconomics. (2009) 27:465–77. 10.2165/00019053-200927060-00003 19640010

[17] Buxton KL Roberts CM Buckingham RJ Pursey N Stone RA. Palliative care service provision for chronic obstructive pulmonary disease patients: results from the 2008 national chronic obstructive pulmonary disease audit. In: Winter Meeting of the British-Thoracic-Society London (2008).

[18] Liu G Hu S Wu J Wu J Dong Z Li H. China Guidelines for Pharmacoeconomic Evaluations. Beijing: China Market Press (2020).

[19] Calzetta L Cazzola M Matera MG Rogliani P. Adding a LAMA to ICS/LABA therapy: a meta-analysis of triple therapy in COPD. Chest. (2019) 12:16. 10.1016/j.chest.2018.12.016 30660781

[20] Lipson DA Barnhart F Brealey N Brooks J Criner GJ Day NC. Once-daily single-inhaler triple versus dual therapy in patients with COPD. N Engl J Med. (2018) 378:1671–80. 10.1056/NEJMoa1713901 29668352

[21] Lipson DA Barnacle H Birk R Brealey N Locantore N Lomas DA. FULFIL trial: once-daily triple therapy for patients with chronic obstructive pulmonary disease. Am J Respirat Crit Care Med. (2017) 438:449OC. 10.1164/rccm.201703-0449OC 28375647

[22] Schroeder M Benjamin N Atienza L Biswas C Ismaila AS. Cost-effectiveness analysis of a once-daily single-inhaler triple therapy for patients with chronic obstructive pulmonary disease (COPD) using the FULFIL trial: a Spanish perspective. Int J COPD. (2020) 15:1621–32. 10.2147/COPD.S240556 32764908 PMC7360413

[23] Papi A Vestbo J Fabbri L Corradi M Prunier H Cohuet G. Extrafine inhaled triple therapy versus dual bronchodilator therapy in chronic obstructive pulmonary disease (TRIBUTE): a double-blind, parallel group, randomised controlled trial. Lancet. (2018) 2018:1076. 10.1016/S0140-6736(18)30206-X 29429593

[24] Martinez FJ Bourne E Ballal S Darken P Aurivillius M Dorinsky P. Budesonide/glycopyrrolate/formoterol fumarate metered dose inhaler improves exacerbation outcomes in patients with COPD without a recent exacerbation history: a subgroup analysis of KRONOS. Dovepress. (2020) 2020:S286087. 10.2147/COPD.S286087 33542624 PMC7851632

[25] Kohansal R Martinez-Camblor P Agustí A Buist AS Mannino DM Soriano JB. The natural history of chronic airflow obstruction revisited. Am J Respirat Crit Care Med. (2009) 180:3–10. 10.1164/rccm.200901-0047OC 19342411

[26] Holly McCabe BG Amanj K Katie J Sean M-S Janey L Simon H. Prescribing trends of inhaler treatments for asthma and chronic obstructive pulmonary disease within a resource-constrained environment in the Scottish national health service: findings and implications. Expert Rev Respirat Med. (2019) 2019:1624528. 10.1080/17476348.2019.1624528 31189394

[27] Abdulsalim S Unnikrishnan MK Mohan MK Alrasheedy AA Godman B Morisky DE. Structured pharmacist-led intervention programme to improve medication adherence in COPD patients: a randomized controlled study. Res Soc Admin Pharma. (2017) 2017:S1551741117308100. 10.1016/j.sapharm.2017.12.008 29104008

[28] Abdulsalim S Unnikrishnan MK Manu MK Alsahali S Alfadl AA. Impact of a clinical pharmacist intervention on medicine costs in patients with chronic obstructive pulmonary disease in India. PharmacoEconomics. (2019) 4:172. 10.1007/s41669-019-0172-x PMC7248138 31368087

[29] Ms A Ds B Nr C Am D Sz E Kn C. Cost-effectiveness analysis of a single-inhaler triple therapy for patients with advanced chronic obstructive pulmonary disease (COPD) using the FULFIL trial: a UK perspective. Respirat Med. 1:100008. 10.1016/j.yrmex.2019.100008

[30] Woo LL Smith HE Sullivan SD. The economic burden of chronic obstructive pulmonary disease in the Asia-Pacific Region: a systematic review. Value Health Regional Issues. (2019) 18:121–31. 10.1016/j.vhri.2019.02.002 31051330

