# Supplementary material for: Cost-Effectiveness Analysis of Triple Combination Preparations in the Treatment of Moderate-to-Severe Chronic Obstructive Pulmonary Disease
Source: Front Public Health. 2021 Jul 28;9:713258. doi: 10.3389/fpubh.2021.713258 (PMC8355539; doi:10.3389/fpubh.2021.713258)
Supplement: Supplementary file 1 [file Table_1.docx]

**Appendix Ⅰ. Population status by age and sex (Nov 1, 2017,to Oct 31, 2018)**

| **Age** | **Mortality rate(‰)** |
| --- | --- |
| Total | 5.87 |
| 0-4 | 1.11 |
| 5-9 | 0.21 |
| 10-14 | 0.21 |
| 15-19 | 0.24 |
| 20-24 | 0.43 |
| 25-29 | 0.44 |
| 30-34 | 0.65 |
| 35-39 | 0.91 |
| 40-44 | 1.36 |
| 45-49 | 2.27 |
| 50-54 | 3.59 |
| 55-59 | 4.73 |
| 60-64 | 8.19 |
| 65-69 | 12.99 |
| 70-74 | 21.08 |
| 75-79 | 40.77 |
| 80-84 | 68.62 |
| 85-89 | 110.14 |
| 90+ | 195.72 |
