# Supplementary material for: Cost-Effectiveness Analysis of Triple Combination Preparations in the Treatment of Moderate-to-Severe Chronic Obstructive Pulmonary Disease
Source: Front Public Health. 2021 Jul 28;9:713258. doi: 10.3389/fpubh.2021.713258 (PMC8355539; doi:10.3389/fpubh.2021.713258)
Supplement: Supplementary file 2 [file Table_2.docx]

**Appendix Ⅱ. COPD Patients Characteristics and Efficacy results（FULFIL）**

FULFIL enrolled patients with COPD aged ≥40 years defined as being in Gold Initiative for Chronic Obstructive Lung Disease group D: i.e. with (a) FEV1 <50% and COPD Assessment TestTM (CAT) score 10, or (b) FEV1 50% to <80% and a CAT score 10, and either 2 moderate exacerbations in the past year or 1 severe exacerbation in the past year.

SGRQ results demonstrated there was a difference of -2.2 (95% CI -3.5, -1.0; p < 0.001) in change from baseline in SGRQ Total score at Week 24 for FF/UMEC/VI versus BUD/FOR in FULFIL. A larger proportion of patients in the FF/ UMEC/VI group (50%) experienced a clinically meaningful improvement from baseline in SGRQ Total score at Week 24, compared with the BUD/FOR group (41%) .

Trough SGRQ Total score responses in FULFIL.

|  | **China subgroup** | | **Non-China subgroup** | |
| --- | --- | --- | --- | --- |
|  | **FF/UMEC/VI  100/62.5/25 μg (n = 32)** | **BUD/FOR  400/12 μg (n = 29)** | **FF/UMEC/VI  100/62.5/25 μg (n = 879)** | **BUD/FOR  400/12 μg (n = 870)** |
| SGRQ Total score | n = 29* | n = 28* | n = 817* | *n* = 763* |
| LS mean at Week 24 (95% Cl) | 45.6 (40.7, 505) | 50.9 (45.9, 55.9) | 44.7 (43.7, 45.6) | 46.7 (45.8, 47.7) |
| LS mean change from baseline (95% Cl) | -5.6 (-10.5, -0.7) | -0.3 (-5.4, 4.7) | -6.6 (-7.5, -5.6) | -45 (-5.4, -3.5) |
| FF/UMEC/VI vs BUD/FOR difference (95% Cl) | -5.3 (-12.3, 1.7) | | -2.1 (-3.4, -0.8) | |
| p-value | 0.14 | | 0.002 | |
| Proportion of SGRQ responders at Week 24* | n = 32* | n = 29* | n = 872* | n = 864* |
| Responders, n (%) | 14 (44) | 9(31) | 434 (50) | 359 (42) |
